# Effects of sodium ferulate for injection on anticoagulation of warfarin in rats in vivo

**DOI:** 10.1186/s12906-024-04389-2

**Published:** 2024-02-14

**Authors:** Yue Zhao, Chunjuan Yang, Yan Liu, Mengnan Qin, Jiahui Sun, Gaofeng Liu

**Affiliations:** 1 Department of Pharmacy, The Second Affiliated Hospital, Harbin Medical University, The Heilongjiang Key Laboratory of Drug Research, Harbin, 150086 P.R. China; 2 College of Pharmacy, Harbin Medical University, Harbin, 150086 P.R. China

**Keywords:** Herb-drug interactions, Sodium ferulate for injection, Warfarin, Anticoagulation

## Abstract

**Background:**

Herb-drug interactions may result in increased adverse drug reactions or diminished drug efficacy, especially for drugs with a narrow therapeutic index such as warfarin. The current study investigates the effects of sodium ferulate for injection (SFI) on anticoagulation of warfarin from aspects of pharmacodynamics and pharmacokinetics in rats and predicts the risk of the combination use.

**Methods:**

Rats were randomly divided into different groups and administered single- or multiple-dose of warfarin (0.2 mg/kg) with or without SFI of low dose (8.93 mg/kg) or high dose (26.79 mg/kg). Prothrombin time (PT) and activated partial thromboplastin time (APTT) were detected by a blood coagulation analyzer, and international normalized ratio (INR) values were calculated. UPLC-MS/MS was conducted to measure concentrations of warfarin enantiomers and pharmacokinetic parameters were calculated by DAS2.0 software.

**Results:**

The single-dose study demonstrated that SFI alone had no effect on coagulation indices, but significantly decreased PT and INR values of warfarin when the two drugs were co-administered (*P* < 0.05 or *P* < 0.01), while APTT values unaffected (*P* > 0.05). *C*_max_ and AUC of R/S-warfarin decreased but *CL* increased significantly in presence of SFI (*P* < 0.01). The multiple-dose study showed that PT, APTT, INR, and concentrations of R/S-warfarin decreased significantly when SFI was co-administered with warfarin (*P* < 0.01). Warfarin plasma protein binding rate was not significantly changed by SFI (*P* > 0.05).

**Conclusions:**

The present study implied that SFI could accelerate warfarin metabolism and weaken its anticoagulation intensity in rats.

## Background

Herbal medicines are widely used as complementary and alternative therapy in clinical practice worldwide. The combined utilization of herbs and drugs is common, but herb-drug interactions may induce unfavorable clinical outcomes, especially for the drugs with a narrow therapeutic index such as warfarin [1, 2].

**Fig. 1:**
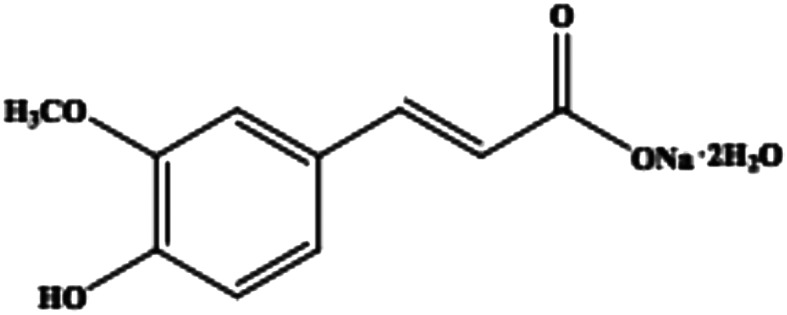
Chemical structure of sodium ferulate

Sodium ferulate injection (SFI) is an important complementary and alternative therapy for patients with vascular diseases including coronary heart diseases, atherosclerosis, cerebrovascular diseases, glomerular diseases, and vasculitis [3–5]. Ferulic acid (4-hydroxy-3-methoxy cinnamic acid, FA), an organic monophenolic phytochemical, is the major active ingredient of SFI. FA is present in many herbal medicines such as *Ligusticum chuanxiong* Hort., *Angelica sinensis* (Olive.) Diels, *Equisetum hyemale* L., *Cimicifugae heracleifolia* Kom., *Picrorhiza scrophulariiflora* Pennell, *Lycium chinense* Mill. and *Ziziphus jujuba* Mill., etc. [5, 6]. Sodium ferulate (Fig. 1), the sodium salt of FA, is mostly used as herbal product due to its high solubility. FA is reported to have a wide range of pharmacological effects including anti-oxidation, anit-hyperlipidemia, anti-atherosclerosis and anti-thrombotic effects [4, 7, 8]. Li et al. explored the influence of sodium ferulate in myocardial ischemic rats by intravenously injecting rats with sodium ferulate. The results showed sodium ferulate can inhibit ischemia reperfusion injury and improve left ventricular remodeling of rats due to its antioxygenation effects and upregulation of miR-133a which has anti-hypertrophy and anti-fibrosis functions [9]. Sodium ferulate can also protect cardiac function of diabetes rats by increasing superoxide dismutase activities and nitric oxide levels and inhibiting oxidative stress both in plasma and myocardium [10]. FA has the potential to prevent and treat liver fibrosis via inhibition of transforming growth factor -β1/Smad pathway in SD rats and human hepatic stellate cell line LX-2 [11]. A study on Institute of Cancer Research mice proved that FA can inhibit platelet aggregation induced by platelet agonists including adenosine diphosphate, thrombin, collagen, and arachidonic acid in a dose-dependent manner [12].

The use of SFI for the treatment of coronary heart disease has been reported in the literatures and presented to be efficient [13–16]. Moreover, the meta-analysis of 36 studies (with 3207 patients) shows that SFI combined with conventional drugs can improve clinical effectiveness for patients with coronary heart disease [17]. As SFI becomes increasingly used, it is necessary to study its interactions with other drugs, especially in combination with high-risk drugs with narrow therapeutic windows such as warfarin to avoid adverse herb-drug interactions. Warfarin can be prescribed to patients with vascular diseases that increase the likelihood of thromboembolic events. The combined effect of SFI and warfarin has not been studied yet, and the interaction between them remains unknown.

Warfarin has been the most commonly prescribed oral anticoagulants worldwide ever since its first approval in 1954 [18]. Even with the spread of novel oral anticoagulants, warfarin is still irreplaceable in antithrombotic therapy, especially in valvular atrial fibrillation (AF) patients with prosthetic heart valves or with rheumatic mitral stenosis [19, 20]. Warfarin is composed of two optically active isomers, R-warfarin and S-warfarin, which are metabolized by different cytochrome P450 (CYP450) enzymes. S-warfarin is mainly metabolized by CYP2C9 whereas R-warfarin is mainly metabolized by CYP1A2 and CYP3A4 [21, 22]. A major challenge for warfarin usage is its narrow therapeutic window, with an effective concentration range of 1.8–2.6 µg/mL, which is associated with adverse events including bleeding events due to over-anticoagulation or thrombotic disorders due to under-anticoagulation [23–26]. Warfarin is susceptible to various exogenous factors including concomitant drugs and has the potential to interact with many drugs, medicinal plants and food, which makes close monitoring more necessary [27]. International normalized ratio (INR), which quantifies coagulation activity provides an appropriate way to monitor effectiveness, safety, and anticoagulation management of warfarin. The optimal INR target of warfarin is generally between 2.0 and 3.0. When INR < 2.0, the therapeutic effect may not be effectively achieved, and thrombosis may occur. When INR > 4.0, patients are at increased risk of over-anticoagulation even bleeding [20, 28].

Both SFI and warfarin have anti-thrombotic pharmacological effects and have been widely used in patients with relative vascular diseases. CYP450 isoenzymes involved in warfarin metabolism mainly include CYP1A2, CYP2C9 and CYP3A4, while FA has an inducing effect on the activity of CYP2C9 and CYP3A4 [29]. Therefore, SFI may influence effectiveness of warfarin by affecting its pharmacodynamics (PD) and pharmacokinetics (PK) when co-administered.

Herb-drug interactions call for attention and research for better treatment and preventing potential adverse events. To better understand herb-drug interactions and relative mechanisms, murine experiments are often used in researches. Some researches have been conducted concerning SFI-drugs or herb-warfarin interactions [5, 30]. In streptozotocin-induced diabetic mice, combination of FA with metformin can significantly reduce blood sugar level over either individual treatment [31]. *Ginkgo biloba* L. extract, a popular herbal ingredient which is widely applied for peripheral arterial diseases and cognitive dysfunction, can decrease warfarin effectiveness via induction of hepatic CYPs by its active ingredient bilobalide in mice [32]. Shenxiong glucose injection is the preparation containing water extracts of *Salvia miltiorrhiza* Bge. and ligustrazine hydrochloride. Animal studies showed that Shenxiong glucose injection could improve the blood concentration of warfarin by inhibiting CYP2C11 in rats [33]. Herba erigerontis injection could increase prothrombin time (PT) and INR values of rats co-administered with warfarin and decrease plasma protein binding rate of warfarin in rats [34].

This article systematically studied the effect and mechanism of SFI on the anticoagulation of warfarin from the perspectives of PD and PK to clarify whether and how SFI influences the anticoagulation intensity of warfarin, which has not been reported before. The current study would provide a foundation to predict risks for the combined use of SFI and warfarin and thereby improve medical therapeutic effect and safety.

## Methods

### Chemicals and reagents

SFI was supplied by Harbin Medisan Pharmaceutical Co., Ltd. (Harbin, China). Warfarin was obtained from Shanghai Xinyi Jiufu Co., Ltd (Shanghai, China). Standards of warfarin sodium and methyclothiazide were purchased from National Institutes for Food and Drug Control (Beijing, China). R/S-warfarin were supplied by Toronto Research Chemicals (Toronto, Canada). Reagents of PT and activated partial thromboplastin time (APTT) were provided by Shanghai Sun Biological Technology Co., Ltd (Shanghai, China). Methanol and acetonitrile were chromatographic grade.

### Experimental animals

Male Wistar rats weighing 200 ± 20 g were supplied by the Animal Center of the Second Affiliated Hospital of Harbin Medical University (Harbin, China), which was accredited by the Institutional Animal Care and Use Committee (IACUC). All procedures complied with the National Society for Medical Research and Guidelines for the Care and Use of Laboratory Animals. Rats were raised in standard cages under 12:12 h light-dark cycle with sufficient food and water. After the last blood collection procedure, rats were injected sodium pentobarbital (120 mg/kg) by tail vein for euthanasia.

### Animal treatment of single-dose warfarin administration

Forty-eight Wistar rats were randomly divided into six groups with eight animals in each group: blank control group, low dose SFI group, high dose SFI group, warfarin control group, warfarin + low dose SFI group, warfarin + high dose SFI group. Rats in the blank control group and warfarin control group were intraperitoneally injected with normal saline for 14 consecutive days, while rats in low dose SFI group, high dose SFI group, warfarin + low dose SFI group and warfarin + high dose SFI group were intraperitoneally injected SFI (8.93 mg/kg or 26.79 mg/kg) for 14 days respectively. On day 8, the rats in the warfarin control group, warfarin + low dose SFI group and warfarin + high dose SFI group were treated with warfarin by oral gavage (0.2 mg/kg), and rats in the blank control group, low dose SFI group and high dose SFI group were given an equal volume of normal saline, respectively.

### Animal treatment of multiple-dose warfarin administration

Wistar rats were randomly divided into warfarin control group, warfarin + low dose SFI group, warfarin + high dose SFI group with eight rats in each group and twenty-four rats totally. All rats were given warfarin (0.2 mg/kg) by oral gavage for 8 consecutive days. Rats in warfarin + low dose SFI group and warfarin + high dose SFI group were intraperitoneally injected SFI (8.93 mg/kg, 26.79 mg/kg) for 4 consecutive days from the 5th day, respectively, and rats in the warfarin control group were administrated normal saline with an equal volume by intraperitoneal injection.

### Animal treatment of the effects of SFI on warfarin plasma protein binding rate

Thirty-two Wistar rats were randomly divided into the warfarin control group, warfarin + low dose SFI group, warfarin + medium dose SFI group, warfarin + high dose SFI group. For 8 consecutive days, rats in warfarin control group were given an equal dose of normal saline, and rats in warfarin + low dose SFI group, warfarin + medium dose SFI group, warfarin + high dose SFI group were given SFI (8.93 mg/kg, 17.86 mg/kg, 26.79 mg/kg), respectively. On day 8, rats in all groups were given intragastric administration of warfarin (0.2 mg/kg). 1.5 h later, blood was taken from the orbit and subjected to ultrafiltration or other tests. The concentration of warfarin was measured with ultra-high-performance liquid chromatography-tandem mass spectrometry (UPLC–MS/MS).

### Pharmacodynamic study

In the single-dose study, blood samples (100 µL) were collected from the retinal venous plexus of the four groups of rats at 0, 4, 8, 12, 24, 36, 48, 72, 96, 120, and 144 h after warfarin administration, respectively. In the multiple-dose study, blood samples (100 µL) of rats from the warfarin control group, warfarin + low dose SFI group and warfarin + high dose SFI group were collected before treatment with warfarin every day. All samples were centrifuged at 3000 r/min for 15 min. PT and APTT values were measured with the automatic blood coagulation analyzer (C2002-2, Precil, Beijing, China). INR = (PT_test_/PT_normal_)^ISI^. PT_test_ and PT_normal_ are prothrombin time of drug-containing plasma samples and blank plasma samples respectively, and ISI stands for International Sensitivity Index.

### Pharmacokinetic study of R/S-warfarin by UPLC-MS-MS

In single-dose study, blood samples (200 µL) were collected from rats in relative groups at the following time points after warfarin administration: 0, 0.5, 1, 1.5, 2, 4, 8, 12, 24, 48, 72, 96, 120 and 144 h. In multiple-dose study, blood samples (200 µL) were collected before each dose of warfarin was given. All the samples were analyzed by UPLC-MS/MS which was performed as described with some modifications [35]. Acetic acid (20 µL) and internal standard (IS) methyclothiazide (50 µL) were added to plasma (100 µL), and vortexed, then ethyl acetate (3 mL) was added and centrifuged (3500 r/min, 5 min). The supernatant was transferred and evaporated under a gentle stream of nitrogen at 40℃. The residue was resuspended with 50% acetonitrile-water solution (100 µL) and filtered. 10 µL aliquot was used for each analysis.

### Instrumentations and chromatographic conditions

The chromatographic separation was conducted by Astec CHIROBIOTIC^TM^V chiral columns (5 μm, 2.1 mm × 100 mm) with a mobile phase of 5 mmol/L ammonium acetate (pH 4.02): acetonitrile = 85:15 (*v/v*) at the flow rate of 0.2 mL/min. 5 µL sample was injected and detected for 12 min for every test at room temperature.

The mass spectrometer was coupled with an electrospray ionization source in positive ion mode for mass spectrometry. Conditions were set as follows: capillary voltage of 0.4 KV, cone voltage of 35 V, and nitrogen flow temperature of 400℃ (flow rate: 650 L/h). Quantification was performed by multiple reaction monitoring mode with parameter setting as follows: (1) warfarin: m/z 307.0→161.0, fragmentation voltage 115 eV, collision energy 20 eV, collision cell acceleration voltage 5 V; (2) IS: m/z 357.8→321.9, fragmentation voltage 100 eV, collision energy 8 eV, collision cell acceleration voltage 5 V. Data were collected and analyzed with G6410A software (Agilent).

### Statistical analysis

DAS2.0 software (Chinese Pharmacological Society, Beijing, China) was used to obtain pharmacokinetic parameters including elimination half-life (*t*_1/2_), peak concentration (*C*_max_), area under the concentration-time curve (AUC), and clearance (*CL*). SPSS 20.0 (IBM CorP. New York, USA) was applied to analyze the pharmacodynamic and pharmacokinetic parameters, and the data are expressed as mean ± SD. Data were compared by independent *t* test, *P* < 0.05 was considered significant and *P* < 0.01 was considered highly significant.

## Results

### The effect of repeated dosing with SFI on the pharmacodynamics of single-dose of warfarin

**Fig. 2:**
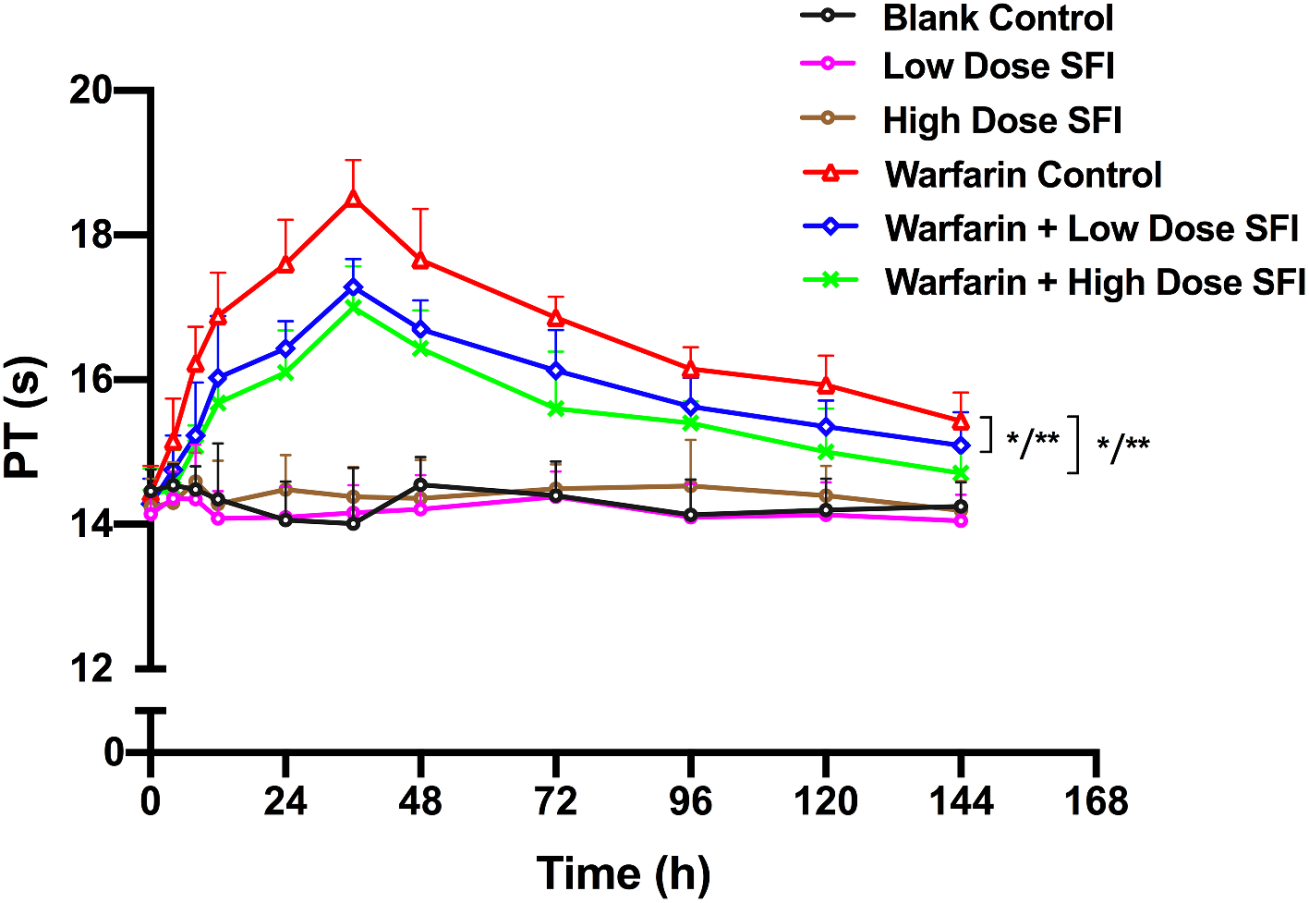
PT-time curves for the effect of SFI on single-dose warfarin pharmacodynamics. Data are expressed as mean ± SD, n = 8 per group. *P < 0.05 vs. Blank Control, **P < 0.01 vs. Blank Control. PT, prothrombin time. SFI, sodium ferulate for injection

**Fig. 3:**
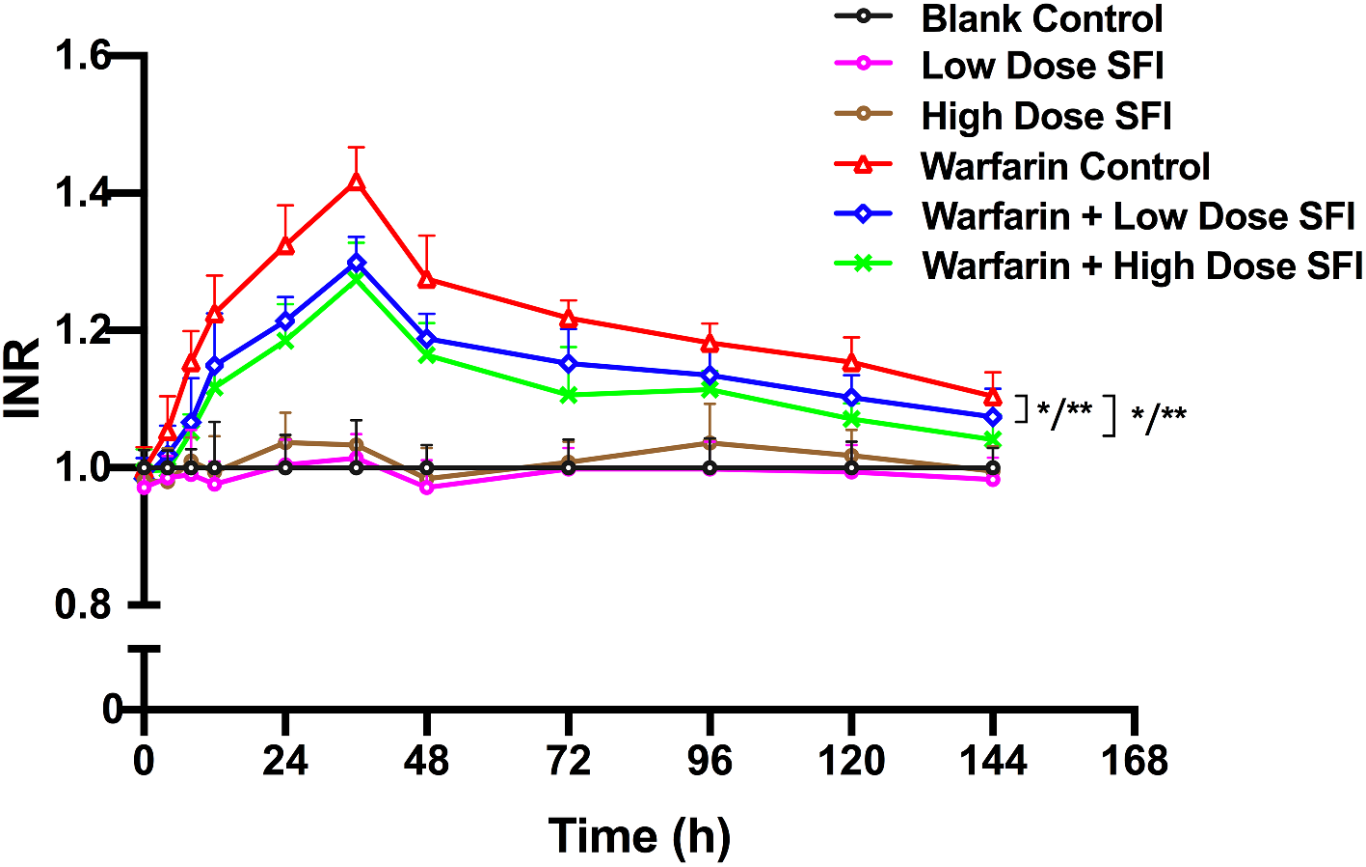
INR-time curves for the effect of SFI on single-dose warfarin pharmacodynamics. Data are expressed as mean ± SD, n = 8 per group. *P < 0.05 vs. Blank Control, **P < 0.01 vs. Blank Control. INR, international normalized ratio. SFI, sodium ferulate for injection

Rats were injected with SFI of low dose (8.93 mg/kg) or high dose (26.79 mg/kg) with or without a single dose of warfarin (0.2 mg/kg). Pharmacodynamics parameters including PT and APTT were measured as described above, and INR values were calculated. PT-time curves and INR-time curves are presented in Figs. 2 and 3. APTT values are shown in Table 1. Compared with the blank control group, PT, APTT, and INR values of the low dose or high dose SFI group were not significantly changed (*P* > 0.05). Compared with the warfarin control group, the PT and INR values of warfarin combined with low dose or high dose SFI group were reduced to varying degrees (*P* < 0.05 or *P* < 0.01), while APTT values showed no significant difference (*P* > 0.05).

**Table 1:** APTT values for the effect of SFI on single-dose warfarin pharmacodynamics

APTT parameters	Blank Control	Low Dose SFI	High Dose SFI	Warfarin Control	Warfarin + Low Dose SFI	Warfarin + High Dose SFI
*E*_max_(s)	25.45	25.90	25.67	36.89	36.24	35.93
*T*_max_(h)	-	24 (12–36)	24 (12–36)	36 (24–48)	36 (24–48)	36 (24–48)

n = 8 per group. APTT, activated partial thromboplastin time. SFI, sodium ferulate for injection

### The effect of repeated dosing with SFI on the pharmacodynamics of multiple dose of warfarin

**Fig. 4:**
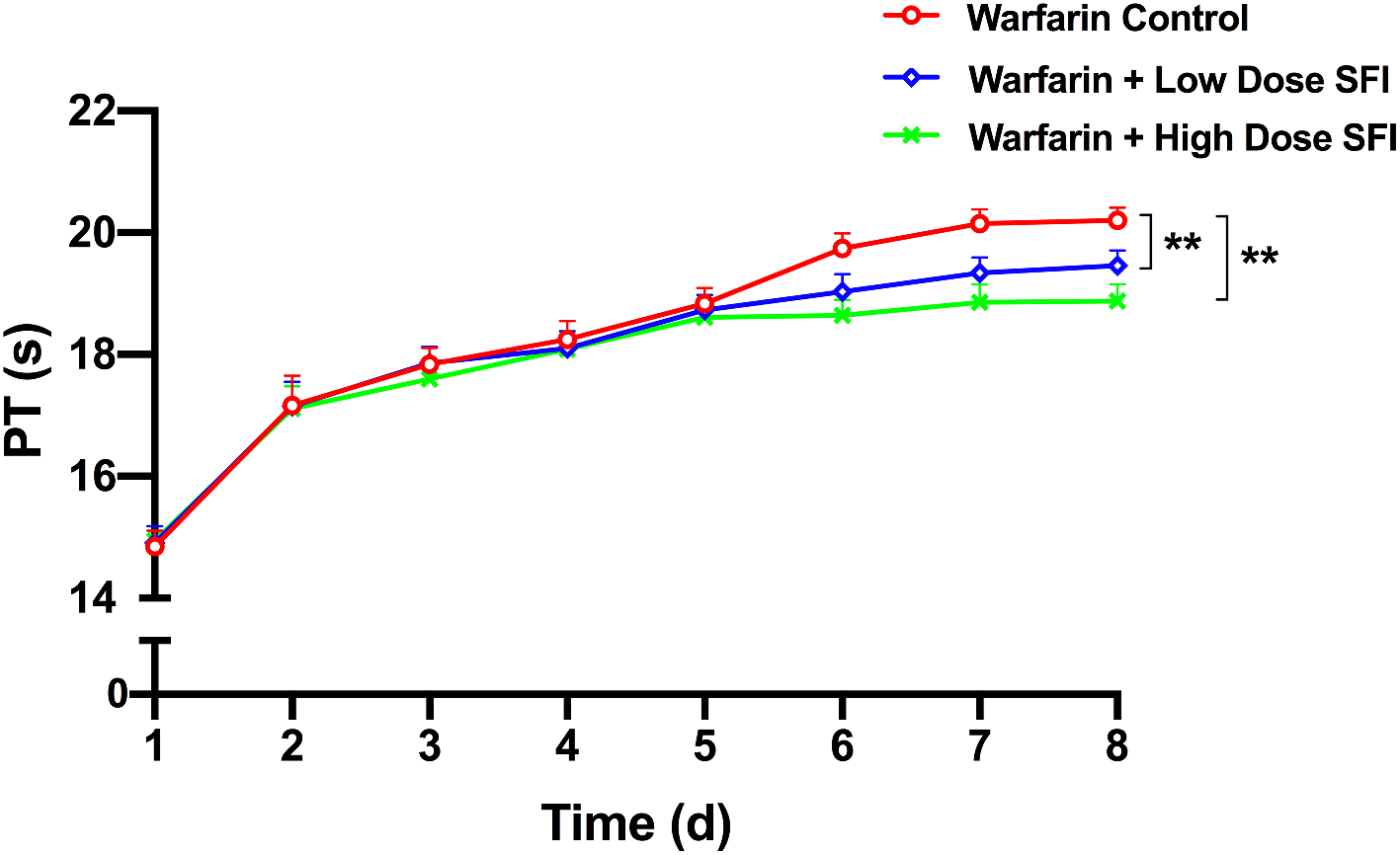
PT-time curves for the effect of SFI on steady-state warfarin pharmacodynamics. Data are expressed as mean ± SD, n = 8 per group. **P < 0.01 vs. Warfarin Control group. PT, prothrombin time. SFI, sodium ferulate for injection

**Fig. 5:**
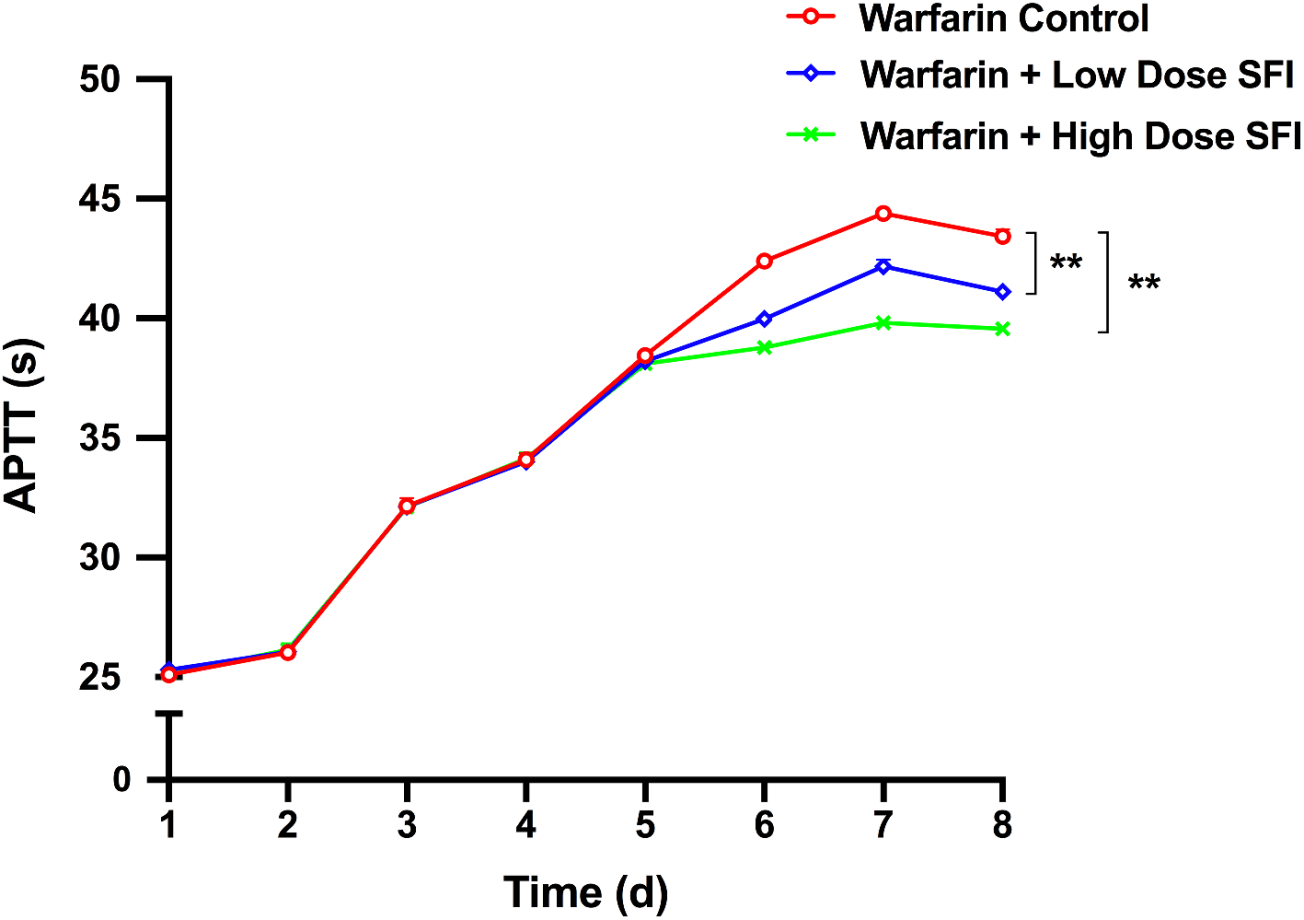
APTT-time curves for the effect of SFI on steady-state warfarin pharmacodynamics. Data are expressed as mean ± SD, n = 8 per group. **P < 0.01 vs. Warfarin Control group. APTT, activated partial thromboplastin time. SFI, sodium ferulate for injection

**Fig. 6:**
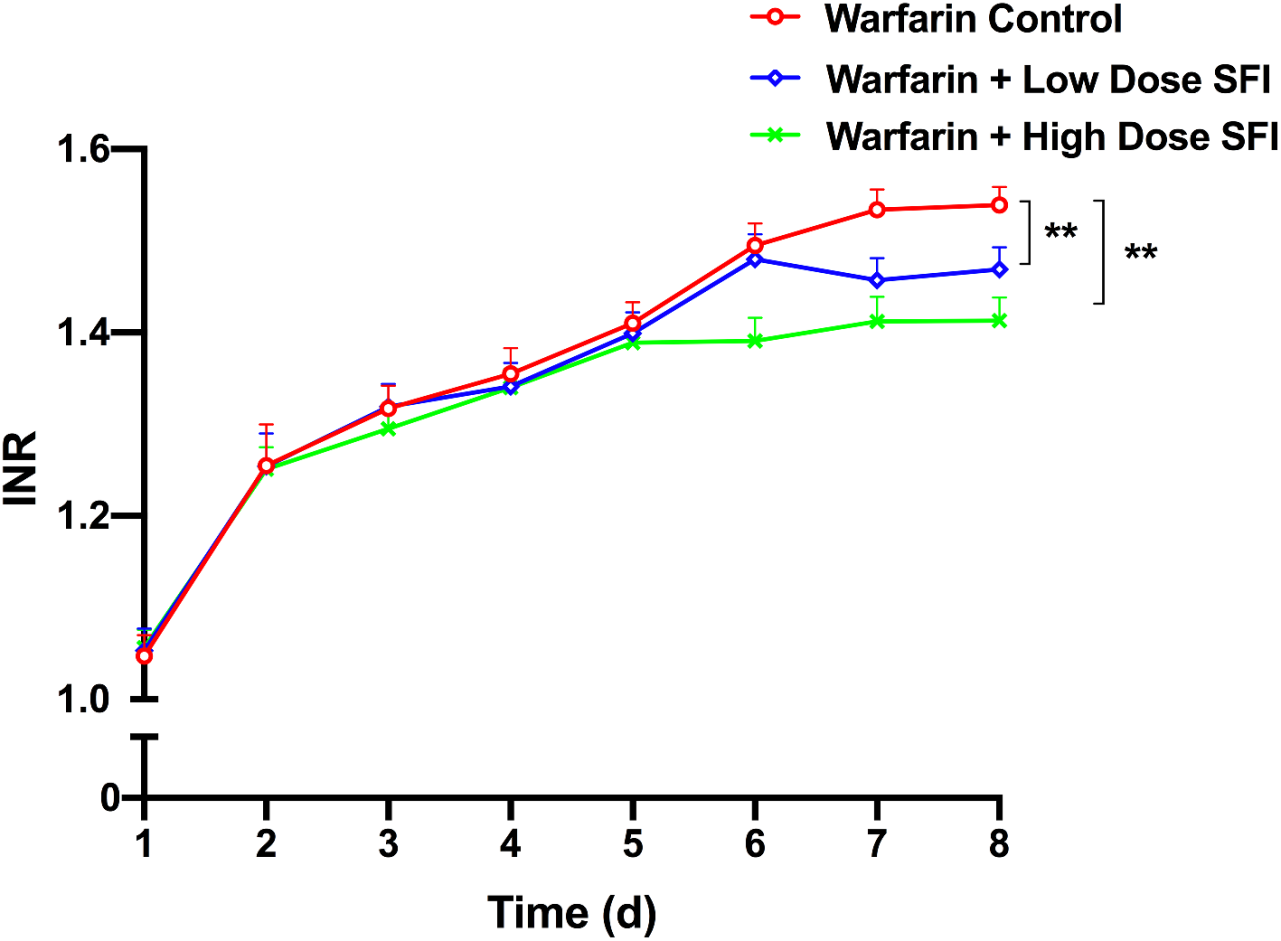
INR-time curves for the effect of SFI on steady-state warfarin pharmacodynamics. Data are expressed as mean ± SD, n = 8 per group. **P < 0.01 vs. Warfarin Control group. INR, international normalized ratio. SFI, sodium ferulate for injection

Rsts were orally given warfarin (0.2 mg/kg) for 8 consecutive days, in which some were intraperitoneally injected with normal saline, SFI of low dose (8.93 mg/kg) or high dose (26.79 mg/kg) for 3 consecutive days from day 5. To measure PT and APTT, rats’ plasma from different groups were then applied to an automated coagulation analyzer. INR values were calculated accordingly. Figures 4, 5 and 6 present PT-time curves, APTT-time curves, and INR-time curves respectively. According to these results, after the blood concentration of warfarin reaches a steady state, low and high dose SFI can significantly decrease PT, APTT and INR values in rats (*P* < 0.01).

### The effect of repeated dosing with SFI on the pharmacokinetics of a single dose of warfarin

**Fig. 7:**
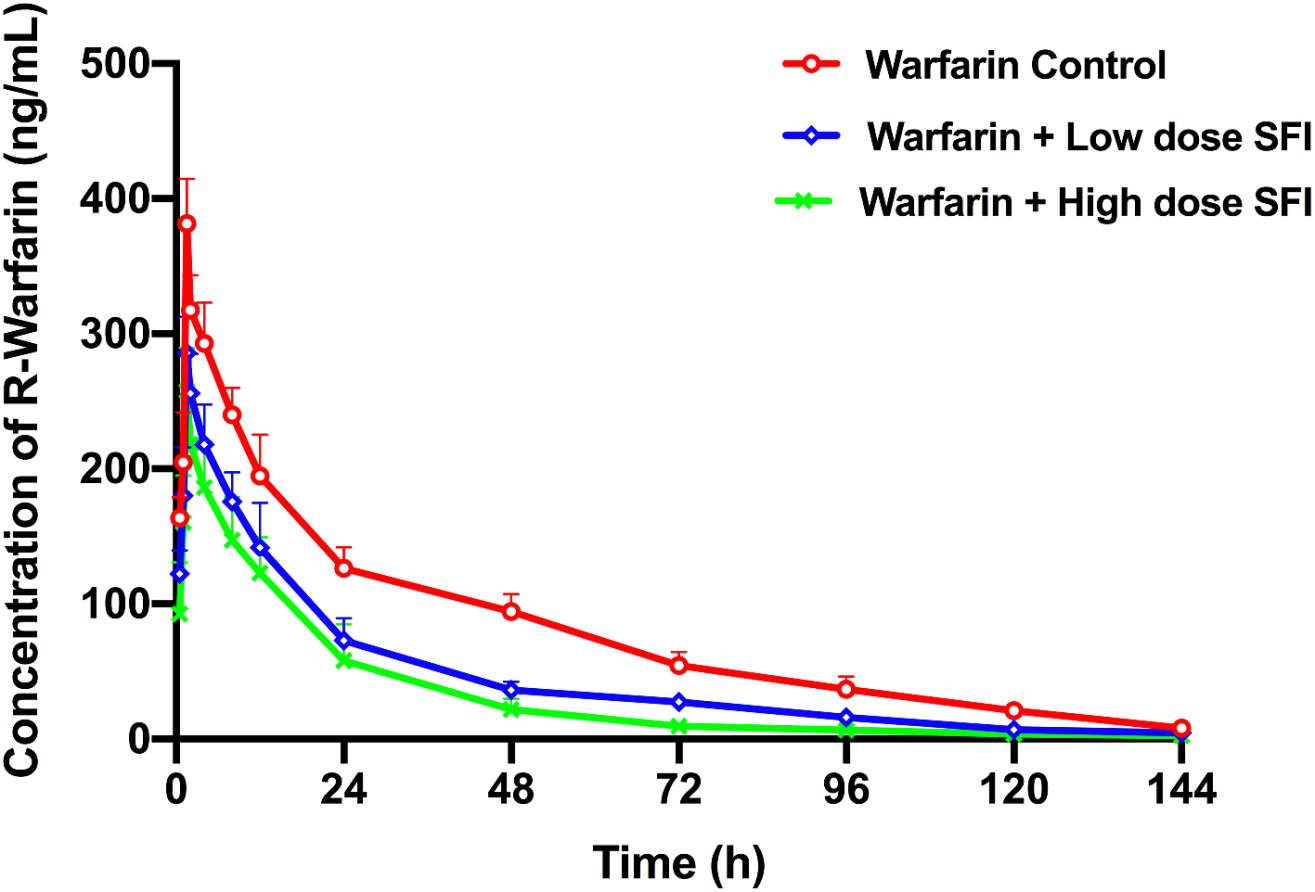
R-warfarin plasma concentration-time curves of the effect of SFI on single-dose warfarin. Data are expressed as mean ± SD, n = 8 per group. SFI, sodium ferulate for injection

**Fig. 8:**
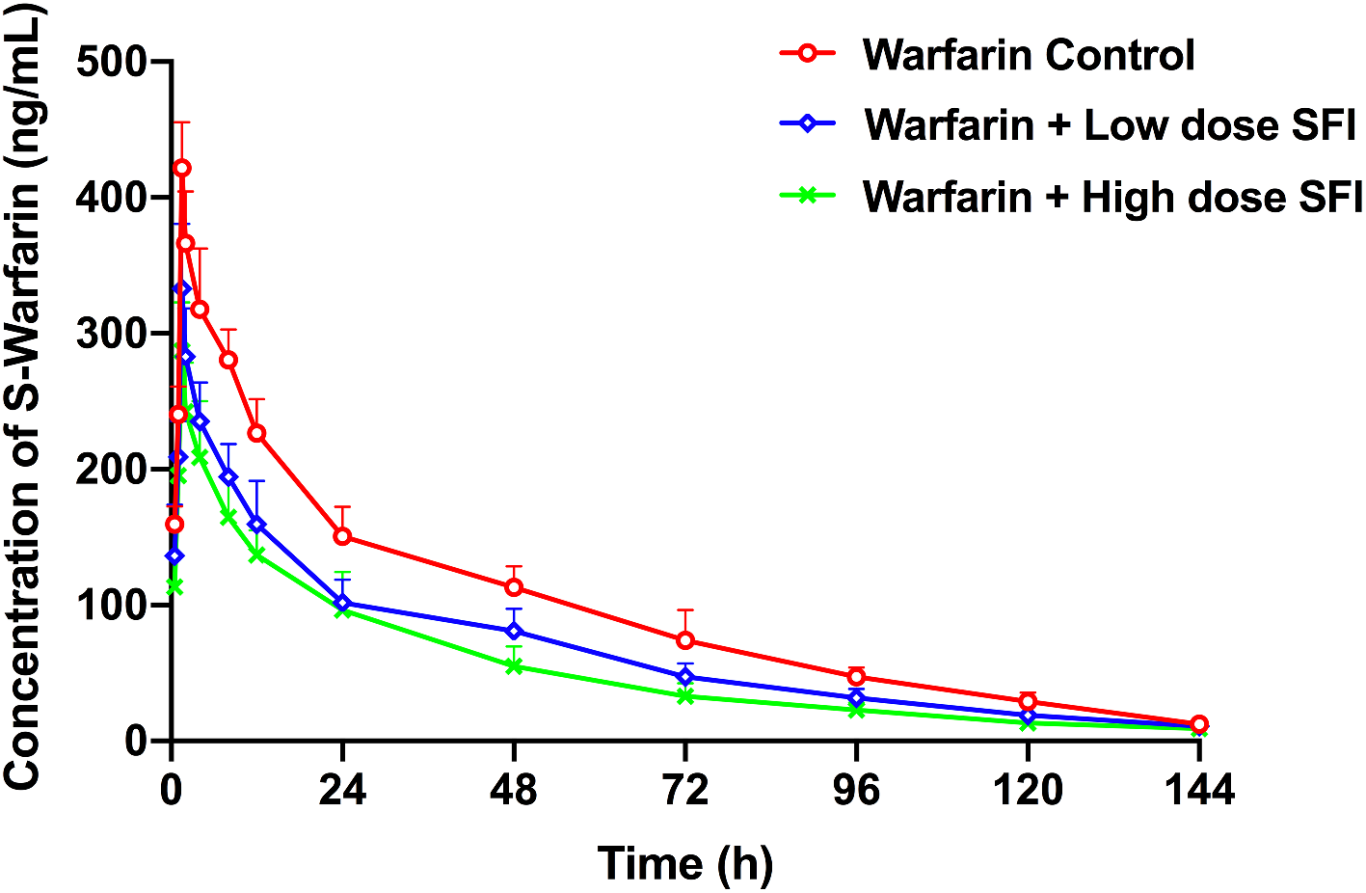
S-warfarin plasma concentration-time curves of the effect of SFI on single-dose warfarin. SFI, sodium ferulate for injection

The pharmacokinetic parameters of R-warfarin were shown in Table 2 and corresponding results of S-warfarin were in Table 3. Figures 7 and 8 depicted plasma concentration-time curves of the two configurations of warfarin. Compared with the warfarin control group, the pharmacokinetic parameter *C*_max_, AUC_0 − t_ and AUC_0−∞_ of R-warfarin in the warfarin + low dose SFI group reduced by 25.10% (*P* < 0.01), 42.55% (*P* < 0.01), 41.29% (*P* < 0.01), *CL* increased by 43.33% (*P* < 0.01), and *t*_1/2_ shortened by 29.67% (*P* < 0.01). The pharmacokinetic parameters *C*_max_, AUC_0 − t_, AUC_0−∞_ of R-warfarin in the warfarin + high dose SFI group reduced by 32.45% (*P* < 0.01), 58.93% (*P* < 0.01), 59.72% (*P* < 0.01), *CL* increased by 86.67% (*P* < 0.01), *t*_1/2_ shortened by 44.31% (*P* < 0.01) (Table 2). The S-warfarin pharmacokinetic parameters *C*_max_, AUC_0 − t_, AUC_0−∞_ in the warfarin + low dose SFI group decreased by 21.03% (*P* < 0.01), 30.79% (*P* < 0.01), 28.49% (*P* < 0.01), *CL* increased by 25.00% (*P* < 0.01), *t*_1/2_ shortened by 8.65% (*P* > 0.05) and S-warfarin pharmacokinetic parameters *C*_max_, AUC_0 − t_, AUC_0−∞_ in the warfarin + high dose SFI group were reduced by 30.28% (*P* < 0.01), 43.44% (*P* < 0.01), 41.57% (*P* < 0.01), *CL* increased by 42.31% (*P* < 0.01), *t*_1/2_ shortened by 14.05% (*P* > 0.05) (Table 3). These data elucidated that SFI could accelerate the metabolism of warfarin enantiomers, especially for R-warfarin in the warfarin + high-dose SFI group.

**Table 2:** The pharmacokinetic parameters of R-warfarin for the effect of SFI on warfarin pharmacokinetics

PK parameter	Warfarin Control	Warfarin + Low Dose SFI	Warfarin + High Dose SFI
*C*_max_ / ng·mL^− 1^	381.62 ± 33.19	285.84 ± 26.74**	257.80 ± 31.83**
*t*_1/2_ / h	36.23 ± 5.77	25.48 ± 2.86**	20.17 ± 3.25**
AUC_0 − t_ / ng·h·mL^− 1^	11465.92 ± 500.71	6586.72 ± 412.23**	4708.94 ± 538.97**
AUC_0−∞_ / ng·h·mL^− 1^	12071.98 ± 603.21	7086.88 ± 511.20**	4863.15 ± 570.77**
*CL*/ mL·h^− 1^·kg^− 1^	0.0017 ± 0.0001	0.0030 ± 0.0003**	0.0043 ± 0.0007**

Data are expressed as mean ± SD, n = 8 per group. **P < 0.01 vs. Warfarin Control. SFI, sodium ferulate for injection

**Table 3:** The pharmacokinetic parameters of S-warfarin for the effect of SFI on warfarin pharmacokinetics

PK parameters	Warfarin Control	Warfarin + Low Dose SFI	Warfarin + High Dose SFI
*C*_max_ / ng·mL^− 1^	421.78 ± 33.75	333.03 ± 47.65**	294.04 ± 31.30**
*t*_1/2_ / h	35.08 ± 3.27	32.05 ± 3.74	30.15 ± 5.26
AUC_0 − t_ / ng·h·mL^− 1^	13926.79 ± 1189.05	9638.18 ± 693.79**	7876.62 ± 840.75**
AUC_0−∞_ / ng·h·mL^− 1^	14500.81 ± 1162.98	10369.60 ± 930.72**	8473.06 ± 677.27**
*CL* / mL·h^− 1^·kg^− 1^	0.0015 ± 0.0001	0.0020 ± 0.0002**	0.0026 ± 0.0004**

Data are expressed as mean ± SD, n = 8 per group. **P < 0.01 vs. Warfarin Control group. SFI, sodium ferulate for injection

### The effect of repeated dosing with SFI on the pharmacokinetics of multiple-dose of warfarin

**Fig. 9:**
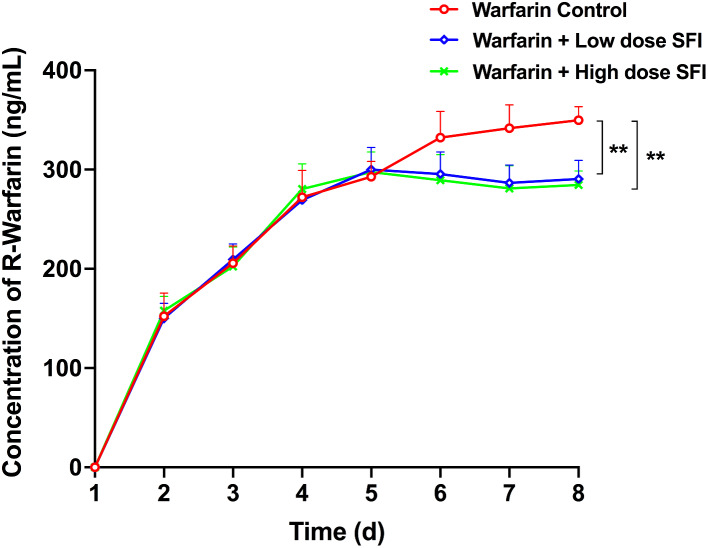
R-warfarin plasma concentration-time curves effect of SFI on steady-state warfarin. Data are expressed as mean ± SD, n = 8 per group. **P < 0.01 vs. Warfarin Control group. SFI, sodium ferulate for injection

**Fig. 10:**
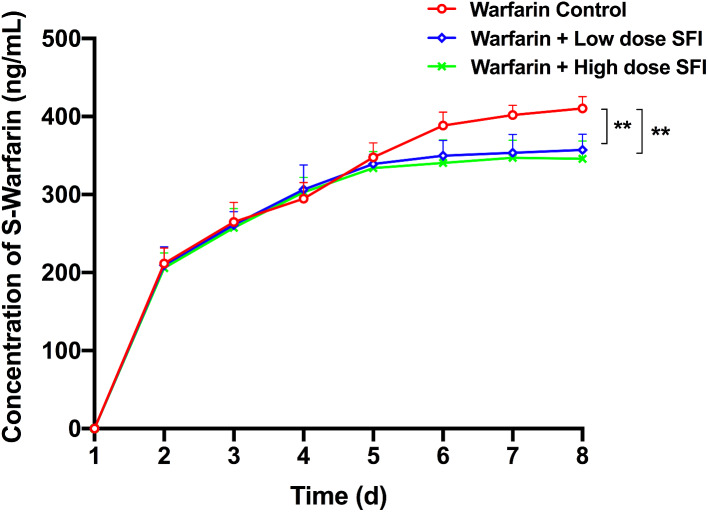
S-warfarin plasma concentration-time curves effect of SFI on steady-state warfarin. Data are expressed as mean ± SD, n = 8 per group. **P < 0.01 vs. Warfarin control group. SFI, sodium ferulate for injection

Plasma concentration-time curves of warfarin enantiomers are shown in Figs. 9 and 10. Compared with warfarin control group, plasma concentrations of R-warfarin decreased by 11.03% (*P* < 0.01), 16.14% (*P* < 0.01) and 16.95% (*P* < 0.01) after low dose SFI was co-administered, or 12.91% (*P* < 0.01), 17.76% (*P* < 0.01), 18.67% (*P* < 0.01) after high dose SFI co-administered at the last three consecutive days (shown as the day of 6, 7, 8 in the Figs. 9 and 10), respectively. Similarly, plasma concentrations of S-warfarin decreased by 9.90% (*P* < 0.01), 12.07% (*P* < 0.01), 12.98% (*P* < 0.01) after low dose SFI was co-administered, and reduced by 12.33% (*P* < 0.01), 13.62% (*P* < 0.01), and 15.74% (*P* < 0.01) after high dose SFI was co-administered. These data indicated that SFI (both low dose and high dose) could promote the metabolism of warfarin in steady-state, mainly manifested by reducing its plasma concentrations.

### The effect of SFI on warfarin plasma protein binding rate

Concentrations of total and free warfarin were measured in the different groups and warfarin plasma protein binding rates were calculated accordingly. As shown in Table 4, warfarin plasma protein binding rates in presence of low-, medium-, and high-dose SFI were 98.03%, 97.95%, and 97.63% respectively, and had no statistical changes. These data implied that SFI had no significant effect on warfarin plasma protein binding rate.

**Table 4:** Warfarin plasma protein binding rate with or without SFI of different doses

Group	Dosage of SFI(mg/kg)	Warfarin plasma protein binding rate (%)
Warfarin Control	**-**	98.30 ± 0.72
Warfarin + Low Dose SFI	8.93	98.03 ± 0.99
Warfarin + Medium Dose SFI	17.86	97.95 ± 1.36
Warfarin + High Dose SFI	26.79	97.63 ± 1.11

Data are expressed as mean ± SD, n = 8 per group. SFI, sodium ferulate for injection

## Discussion

The current study assessed the effects of SFI, a herbal medicine widely used in cardiovascular and cerebrovascular diseases, on warfarin anticoagulation effects from perspectives of PD and PK, and predict the risk of the concomitant use. To our knowledge, this is the first study exploring the SFI-warfarin interaction. Our results showed that SFI can weaken anticoagulation effect of warfarin in rats in vivo and it’s suggested to avoid co-administration of SFI or drugs containing FA to patients receiving warfarin.

The coagulation pathways in the body mainly include endogenous coagulation pathways reflected by APTT and exogenous coagulation pathways reflected by PT [36, 37]. The INR value is calculated according to PT and ISC values. Close monitoring of INR value is needed for patients on warfarin to avoid adverse events or therapeutic failures [38–40]. In this study, APTT, PT and INR were measured to investigate how warfarin’s anticoagulation intensity was impacted by SFI and in which coagulation pathways. The pharmacodynamic results of the single-dose warfarin test showed that compared with the blank control group, PT, APTT, and INR of the rats in the low dose or high dose SFI groups were not significantly different (*P* > 0.05). It has been reported that SFI has various pharmacological effects including anti-platelet aggregation, reducing blood lipid level, protecting vascular endothelium, antioxidant effect and increasing coronary artery flow, ect [6]. Our study reported for the first time that SFI has no effect on coagulation parameters, suggesting that SFI does not treat cardiovascular diseases by changing coagulation factors. The results further clarifies the pharmacological mechanism of SFI, and provides more reference for the rational use of natural products or preparations containing FA to treat cardiovascular disorders in traditional practice.

However, compared with the warfarin control group, the combination of the low dose and high dose SFI reduced PT and INR values (*P* < 0.01), while APTT values were not significantly changed (*P* > 0.05), suggesting that SFI could weaken the anticoagulation of single-dose warfarin in exogenous coagulation pathway. Moreover, pharmacodynamic results of the steady-state warfarin test showed that PT, APTT, and INR values were downregulated (*P* < 0.01) when warfarin reached a steady state and was combined with low dose or high dose SFI. These results indicated that SFI alone had no effect on the coagulation index, but when co-administrated with warfarin, it could weaken the anticoagulant effect of single-dose and steady-state warfarin in rats. SFI exerts anti-thrombotic effects by inhibiting platelet aggregation and release [41], whereas warfarin acts by inhibiting the synthesis of coagulation factors due to its structural resemblance of vitamin K [42]. Different anti-thrombotic mechanism of both drugs can explain why SFI attenuates the effect of warfarin but keeps its own anti-thrombotic effect when not co-administered with warfarin.

Our single-dose and steady-state warfarin pharmacokinetic studies showed that both low and high doses of SFI could accelerate the metabolism of warfarin enantiomers in vivo and weaken its anticoagulant effect. There has been reported that ferulic acid had an induction effect on both CYP3A4 and CYP2C9 transcription activity [29]. Since CYP3A4 and CYP2C9 are the main metabolic enzymes for R-warfarin and S-warfarin, respectively, it is speculated that SFI enhances the metabolism of warfarin by inducing these two enzymes, thus weakening its anticoagulant effect.

Clinically available warfarin is a racemic mixture composed of R-warfarin and S-warfarin in equal proportions, while S-warfarin holds 3–5 times more anticoagulation potency than R-warfarin. Both enantiomers are eliminated via hepatic metabolism by different CYP450 enzymes, of which R-warfarin is mainly metabolized by CYP1A2 and CYP3A4, and S-warfarin by CYP2C9 [43]. Therefore, examining the concentration of warfarin in specific enantiomers helped identify metabolic enzymes changed when warfarin was co-administrated with SFI. Warfarin has a high plasma protein binding rate of 98 − 99%. When co-administered drugs compete with warfarin for the binding, it will increase free concentration of warfarin and bleeding risk [44–46]. Our study showed that the plasma protein binding rate of warfarin did not change significantly after warfarin was combined with low, medium, and high doses of SFI (*P* > 0.05). The plasma protein binding rate of sodium ferulate is low at only 19.4%, so it is less likely to compete with warfarin for the binding of plasma protein [6, 47]. Therefore, the combination of the two drugs will not cause drug interactions due to plasma protein binding.

With the increasing application of SFI in the treatment of cardiovascular and cerebrovascular diseases, its interaction with other drugs besides warfarin should also be concerned and studied. It should also be pointed out that according to the previous reports [13–17], the quality of evidences for the efficacy of SFI in the treatment of ischemic cardiovascular and cerebrovascular diseases is not high, due to the presence of clinical heterogeneity, and imprecision in partial outcome measures. Standard therapy is mostly established on large-scale randomized controlled trials (RCT) of high quality and meta-analyses based on those trials [48, 49]. For instance of antiplatelet therapy, to explore appropriate dose of aspirin (81 mg vs. 325 mg) in patients with established atherosclerotic cardiovascular disease, Jones et al. conducted a RCT enrolling 15,076 patients with a median follow-up of 26.2 months and got the conclusion after rigorous statistical analysis [50, 51]. Antithrombotic Trialists’ Collaboration included 287 randomized trials involving more than 200,000 participants to determine the effects of antiplatelet therapy among patients at high risk of occlusive vascular events [52]. Baigent et al. undertook meta-analyses based on six primary prevention trials and sixteen secondary prevention trials [53]. However, there have been no large-scale RCTs investigating the efficacy of SFI in the treatment of cardiovascular and cerebrovascular diseases, no meta-analysis based on high quality of data as well. Therefore, more high quality evidences of clinical trials for SFI are still needed to conduct to further confirm its efficacy.

## Conclusions

Herb-drug interactions may result in adverse events which is a non negligible health issue demanding extensive recognition and deep research. When SFI is applied for treatment of cardiovascular diseases as a complementary and alternative therapy, it should be noticed the potential interaction with warfarin. Warfarin is widely used in thromboembolic diseases but susceptible to various factors especially drugs, which may limit it to attain therapeutic responses. The results of this study indicate that SFI can weaken the anticoagulant effect of warfarin, which may lead to warfarin therapeutic failure and increase the risk of thrombosis. Therefore, it is suggested that it is necessary to avoid giving preparations with FA to patients taking warfarin. Once co-administrated, more close monitoring is needed, and the dosage of warfarin should be adjusted according to the INR value to ensure the effectiveness of anticoagulant therapy and avoid thromboembolic events. More studies should be conducted to confirm this finding of SFI-warfarin interaction in humans.

## Data Availability

The datasets used and/or analyzed during the current study are available from the corresponding author on reasonable request.

## Abbreviations

SFI: Sodium ferulate injection
PT: Prothrombin time
APTT: Activated partial thromboplastin time
INR: International normalized ratio
AF: Atrial fibrillation
PD: Pharmacodynamics
PK: Pharmacokinetics
FA: Ferulic acid
IS: Internal standard
RCT: Randomized controlled trials
